# Numerical simulation of the tsunamis generated by the Sciara del Fuoco landslides (Stromboli Island, Italy)

**DOI:** 10.1038/s41598-019-54949-7

**Published:** 2019-12-06

**Authors:** A. Fornaciai, M. Favalli, L. Nannipieri

**Affiliations:** Istituto Nazionale di Geofisica e Vulcanologia, Sezione di Pisa, Pisa, Italy

**Keywords:** Natural hazards, Geology, Geophysics, Volcanology

## Abstract

Stromboli volcano (Aeolian Arc, Italy) experiences many mass failures along the Sciara del Fuoco (SdF) scar, which frequently trigger tsunamis of various sizes. In this work, we simulate tsunami waves generated by landslides occurring in the SdF through numerical simulations carried out in two steps: (i) the tsunami triggering, wave propagation and the effects on Stromboli are simulated using the 3D non-hydrostatic model NHWAVE; (ii) generated train waves are then input into the 2D Boussinesq model FUNWAVE-TVD to simulate wave propagation in the Southern Tyrrhenian Sea (STS). We simulated the following scenarios: (i) the tsunami runup, inland inundation and wave propagation at Stromboli triggered by submarine landslides with volumes of 7.1, 11.8, 17.6 and 23.5 × 10^6^ m^3^ and subaerial landslides with volumes of 4.7, 7.1, 11.8 and 35.3 × 10^6^ m^3^; (ii) tsunami propagation in the STS triggered by submarine landslides with volumes of 11.8 and 17.6 × 10^6^ m^3^ and by subaerial landslides with volumes of 7.1 and 35.3 × 10^6^ m^3^. We estimate that the damages of the last relevant tsunami at Stromboli, which occurred in 2002, could have been generated either by a subaqueous failure of about 17.6-23.5 × 10^6^ m^3^ along the SdF or/and a subaerial failure of about 4.7-7.1 × 10^6^ m^3^. The coasts most affected by this phenomenon are not necessarily located near the failure, because the bathymetry and topography can dramatically increase the waves heights locally. Tsunami waves are able to reach the first Stromboli populated beaches in just over 1 minute and the harbour in less than 7 minutes. After about 30 minutes the whole Aeolian Arc would be impacted by maximum tsunami waves. After 1 hour and 20 minutes, waves would encompass the whole STS arriving at Capri.

## Introduction

**Figure 1:**
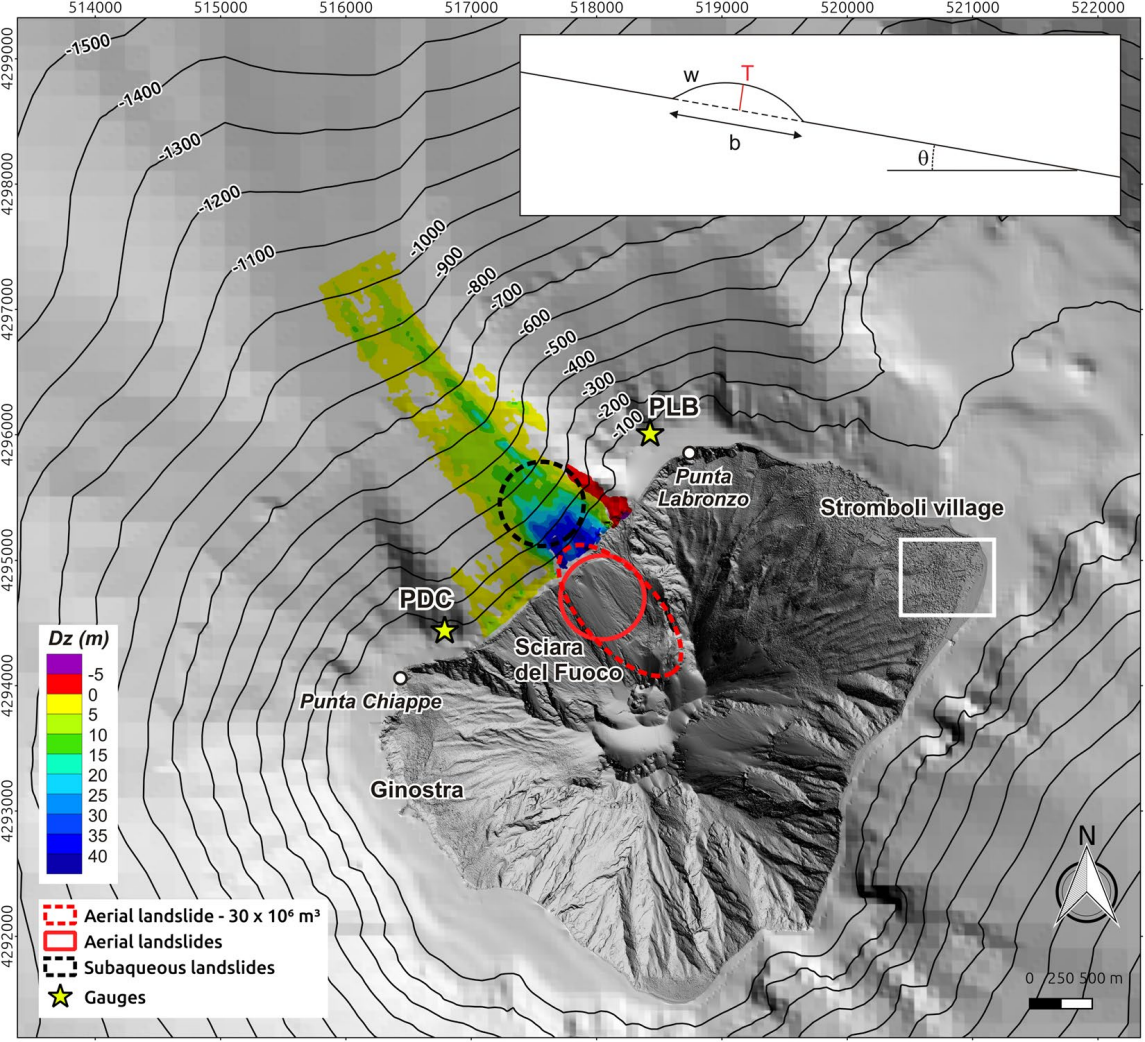
(**a**) Hillshading of Stromboli topo-bathymetric grid with the locations and shape of the simulated landslides based on Fig. 9 showed in Chiocci *et al*.^33^. PDC (Punta Dei Corvi) and PLB (Punta LaBronzo) are the locations of the monitoring gauges managed by the Laboratory of Experimental Geophysics of the Department of Earth Sciences of the University of Florence (http://lgs.geo.unifi.it/). White square shows the location of trenches described in Rosi *et al*.^4^. The inset shows the vertical cross section of the triggering slide (after Ma *et al*.^12^). Map was generated using Quantum GIS 2.8.1 software (https://www.qgis.org).

Stromboli Island is an active volcano characterized by persistent Strombolian activity. This activity is periodically interrupted by flank eruptions or paroxysmal explosions^1,2^, which often contribute in generating large mass failures along the most unstable sector of the volcanic edifice: the Sciara del Fuoco (SdF) depression, on the NW flank of the island (Fig. 1). Landslides at Stromboli can be tsunamigenic^3^. Three tsunami deposits on Stromboli dating back to the Late Middle Ages, of which the oldest was related to a massive landslide, were recently discovered^4^. Since the early 20^th^ century, six tsunamis generated by landslides within the SdF have been documented^1,5,6^. The last relevant tsunami at Stromboli occurred on 30 December 2002 as a consequence of the collapse of a large portion of the SdF, during the 2002–03 eruption^7,8^. The waves caused significant damage to the buildings on the east coast of the island up to an altitude of about 10 m above sea level, caused damage on Panarea and were largely detected along the Southern Tyrrhenian Sea (STS) coasts^8,9^. Simulation of tsunami waves resulting from the collapse of volcano portions is crucial in the STS where numerous active volcanoes (i.e. Stromboli, Vulcano, Lipari, Ischia and Marsili) are concentrated. In addition, the paucity of real data necessary to “instruct” the tsunami detection systems, as the two gauges off the SdF designed for real-time monitoring (http://lgs.geo.unifi.it), makes simulated data particularly valuable. This was dramatically noticeable during the 3 July 2019 and 28 August 2019 Stromboli’s paroxysms, when the two gauges recorded small tsunamis, reasonably due to the entrance of pyroclastic flows into the sea in front of the SdF^10^. Simulating a landslide-generated tsunami is particularly complex because it is necessary to take into account the landslide dynamics (aerial or submarine), its interaction with the water table, the wave generation and propagation and the runup on the coast. As a result, various numerical codes with different approaches and level of complexity have been developed to model landslide-generated tsunamis^11^. 2002 landslide-generated tsunami at Stromboli was simulated by Tinti *et al*.^7^ in two steps: (1) the landslide motion was computed using a Lagrangian block model to compute the instantaneous rate of the vertical displacement of the bottom surface caused by the motion of the underwater slide; (2) the output of step 1 was used as boundary conditions for a wave-propagation model based on shallow water equations and solved by means of a finite-element technique. Tinti *et al*.^7^ model is a depth average code that technically does not permit the computation of water flooding and water runup heights, but only the maximum water elevation at the coast. Therefore, Tinti *et al*.^7^ were not able to produce inland water penetration maps, and runups were approximated as the maximum water elevation at the coast, underestimating or even neglecting many processes such as positive interference of fronts, coastal morphology favouring confluence and transient piling up of incoming water masses and so on.

In this work, we used the non-hydrostatic three-dimensional (sigma-layer) model NHWAVE^12^ to generate and propagate tsunami waves caused by various SdF flank failures, both aerial and subaqueous, as well as to estimate their impact on Stromboli. A fully nonlinear dispersive Boussinesq long wave model (FUNWAVE-TVD)^13^, initialized with the NHWAVE outputs, was used to simulate the propagation of tsunami waves in the STS. FUNWAVE-TVD was used because it is much faster than NHWAVE, allowing to considerably reduce the computational time for the larger computational grid. The novelty and advancements introduced by using NHWAVE and FUNWAVE-TVD for simulating tsunami at Stromboli are: (1) the possibility to compare the results of Tinti *et al*.^7^ with those obtained with different codes; (2) NHWAVE is not a simple depth average code, but a 3D code that allows a number of vertical levels; (3) NHWAVE can calculate directly inland penetration and runup; (4) NHWAVE and FUNWAVE-TVD are freely available codes; (5) NHWAVE and FUNWAVE-TVD are used by different research groups and have been benchmarked. This work is aimed at providing a more comprehensive tsunami impact assessment at Stromboli and in all STS coastlines by simulating for the first time several landslide scenarios at different scales of observation. Moreover, compared to previous works, we extend the study area as far as Capri to the north and Mondello to the west and we use updated topo-bathymetric data.

## Tsunami Simulation

### Computational grid

Bathymetry and topography play a crucial role in the tsunami waves dynamics and impact on the coastal areas. Bathymetry strongly influences tsunami propagation, both driving waves along preferential direction or creating natural dams able to break up the waves energy^7,14^. Coastal bathymetry and local inland topography controls the tsunami runup and inland penetration. For these reason, topo-bathymetric computational grid must be as updated as possible and must have the best possible resolution. Computational grids for tsunami simulation were here built by elaborating and merging data from different sources, with various coverage and acquisition time. The topography of Stromboli was acquired by using an airborne Light Detection and Ranging (LiDAR) system. The LiDAR survey was carried out in July 2010 by using an Optech ALTM Gemini laser altimeter^15^. The mean point density was 1 pts/m^2^ with a nominal horizontal and vertical accuracy of 40 and 15 cm, respectively. TINITALY 10-m digital elevation model^16^ was used as topography for the Southern Tyrrhenian areas, including the Aeolian Arc. Bathymetry covering most of the Southern Italian margins data was provided by the MaGIC project^17^ within the framework of the working group promoted by the Civil Protection Department (DPC) of Italy. MaGIC database provides data with spatial resolution decreasing with the depth, from 50 m to 100 and 200 m moving away from the coast. Bathymetric data from different sources^18,19^ and interpolated as described in Favalli and Pareschi^20^ were used where MaGIC data lacked. Before merging all grids together, the data collected was elaborated in order to correct the mismatching among adjacent grids from different sources and/or survey and artifacts as well as to fill areas with no-data. Two final topo-bathymetric computational grids in WGS84 UTM 33 coordinates were produced: (i) 920 × 660 pixels grid of Stromboli with a cell size of 10 m; (ii) 2959 × 3062 pixels grid of the STS with a cell size of 100 m. The 10 m grid was used as a computational grid for running the NHWAVE simulation of a submarine slide tsunami at Stromboli. In case of subaerial scenarios, the 10 m grid was down-sampled at 20 m and at 30 m to speed up calculation times. The 100 m grid was used as a computational grid for running the FUNWAVE-TVD simulation of the STS.

### Numerical models

Tsunami generation, propagation and runup on Stromboli were simulated by using Non-Hydrostatic WAVE model (NHWAVE)^12^. It provides a numerical solution to the 3D Euler equations for incompressible flows in a terrain and surface-following σ coordinates (i.e., boundary fitted, 3–5 levels in this work). NHWAVE has been validated for highly dispersive landslide tsunami generation by comparing simulated surface elevations with data from laboratory experiments^12,21^. NHWAVE was successfully applied to study tsunami wave generation by submarine landslides^22,23^. NHWAVE can simulate tsunami generation by using three-dimensional rigid underwater landslides, treated as simple bottom movement with idealized shape and behavior^12^. The slide has a nearly elliptical footprint on the slope, with length *b* and width *w*, and vertical cross sections varying according to truncated hyperbolic secant functions in the two orthogonal directions, *ξ* and *η*, with maximum thickness *T* (Fig. 1, inset)^12,21^, which is given by:

1 $$\zeta =\frac{T}{1-\varepsilon }\{\text{sec}{\rm{h}}({k}_{b}\xi ){\text{sec}}h({k}_{w}\eta )-\varepsilon \}$$

where *k*_*b*_ = 2 *C/b*, *k*_*w*_ = 2 *C/w* and *C* = acossh(1/*ε*), with *ε* the truncation parameter. NHWAVE dictates the slide block motion to follow rectilinear trajectories, following the formalism in Enet et Grilli^21^ (cfr. formulas 7–11). The morphology of the SdF’s submarine portion is peculiar having a high-sloped deep depression initially directed northwestern that abruptly turns to north at about 1000 m below sea level (bsl)^24^ (Fig. 1). In order to force the slide to follow the bathymetry, we directly implemented in NHWAVE the equation that governs the center of mass motion parallel to the slope described in Enet et Grilli^21^ (cfr. formula 3), that is:

2 $$({M}_{b}+\Delta {M}_{b})\ddot{s}=({M}_{b}-{\rho }_{w}{V}_{b})(sin\theta -{C}_{n}cos\theta )g-\frac{1}{2}{\rho }_{w}({C}_{F}{A}_{w}-{C}_{D}{A}_{b}){\dot{s}}^{2}$$

where *g* is the gravitational acceleration; Δ*Mb*, *Vb*, *A*_*w*_ and *A*_*b*_ are the slide added mass, volume, wetted surface area and main cross section perpendicular to the direction of motion, respectively; *C*_*n*_ is the basal Coulomb friction coefficient, *C*_*F*_ is the skin friction coefficient and *C*_*D*_ is the form drag coefficient. Equation (2) can be simplified to^21^:

3 $$(\gamma +{C}_{m})\ddot{s}=(\gamma -1)(sin\theta -{C}_{n}cos\theta )g-\frac{1}{2}{C}_{d}\frac{{A}_{b}}{{V}_{b}}{\dot{s}}^{2}$$

where *C*_*m*_ is the added mass coefficient, *C*_*d*_ the global drag coefficient and *γ* is the relative landslide density. Implementation of Eq. 2 inside NHWAVE also allows to simulate the aerial motion, after having accordingly changed the parameters. It is worth to note that in this case the code doesn’t take into account the water splash. NHWAVE is able to calculate maximum inundation area, runup and arrival time on the coasts. To understand which parameters mostly influence the code, a quick sensitivity analysis was performed by comparing the tsunami waveforms obtained varying volume, position, shape and density of the subaqueous triggering landslide. Aside from the starting position and density, which were constrained by volcanological observation (see below), the results show that the volume influences tsunami waves much more than the slide shape.

The STS tsunami propagation was simulated by using FUNWAVE-TVD model^13,25^ initialized with the surface elevation and depth-averaged horizontal velocity fields output by NHWAVE. FUNWAVE-TVD is developed based on the fully nonlinear Boussinesq equations of Chen^26^ and it uses a “total variation diminishing” (TVD) scheme to better model wave breaking dissipation and coastal inundation^27–31^. FUNWAVE-TVD was successfully validated by means of analytical, laboratory, and field benchmarks^32^. FUNWAVE was run by using the NHWAVE solution at t = 217 s on a 100 m pixel size grid, to avoid computational instabilities. This FUNWAVE initialization time allowed for the main waves train leaving Stromboli to be well defined and far enough from the coast to cope with the problems related to the transition from a multi-layer to a depth averaged code. Although FUNWAVE may account for runup and inundation area, a 100-m calculation grid prevented us from obtaining meaningful inundation areas. Hence, we here report just the height of tsunami waves near the Southern Tyrrhenian coasts, taking into account that it is a very rough approximation of the real runup.

### Slide scenarios and 2002 field data

Given that the initial parameters of a triggering landslide at Stromboli are largely unknown and considering the sensitivity analysis performed here, only the variations in the slide volume were modeled. Four slide scenarios were considered for both subaerial and submarine mass failure. Based on volumes inferred by Chiocci *et al*.^33^, the extreme case scenarios hypothesized by Tommasi *et al*.^34^ for the 2002 tsunami and with the aim of identifying tsunami wave impact scenarios in the STS coasts, we simulated tsunamis generated by submarine slides of 7.1, 11.8, 17.6 and 23.5 × 10^6^ m^3^ and subaerial slides of 4.7, 7.1, 11.8 and 35.3 × 10^6^ m^3^. Landslides with these volumes (>10^6^ m^3^) are among the worst-case scenario considered in the definition of the criticality levels for slope instability at Stromboli volcano proposed by Schaefer *et al*.^35^. These levels are based on the intersection between ground displacement monitoring data (the Ground-Based Interferometric Synthetic Aperture Radars, GBInSARs) and the instability scenario based on the results of the slope stability analysis. Moreover, recent studies have shown that landslides of the same order of magnitude as those of 30 December 2002 are always possible and that the conditions of stability of the Sciara del Fuoco have returned to be compatible with those preceding the 2002 landslides^36–39^.

The initial slides were placed as an additional elevation on the grids. Their locations were inferred based on the data collected after the 2002 tsunami at Stromboli^33^ and by considering the geometry imposed by the code, the input volumes and the SdF’s morphology. Aside from the worst case (i.e. 35.3 × 10^6^ m^3^), the slides in all scenarios had a circular footprint with a radius of 670 m (Fig. 1). The 35.3 × 10^6^ m^3^ slide had an elliptical footprint with the major axis (1300 m) following the inclination of SdF and the minor one (600 m) perpendicular to it (Fig. 1). Submarine slides centers coordinates were: x = 517563, y = 4295449 and z = −293 m. Centers coordinates of sub-aerial slides with circular footprints were: x = 518054, y = 4294622 and z = 250 m. Due to its size and footprint, the center coordinates for the 35.3 × 10^6^ m^3^ slide were: x = 518186, y = 4294600 and z = 350 m (Fig. 1). Coordinates are in WGS84, UTM 33 reference system. To account for the different volumes, the slide thickness changed for every scenario, i.e. for the 4.7, 7.1, 11.8, 17.6, 23.5 and 35.3 × 10^6^ m^3^ cases, the maximum thicknesses were respectively 29.9, 45.0, 74.7, 112.0, 194.4 and 149.4 m. Considering the slide as rigid body of volcaniclastic material, a constant density of 2600 kg/m^3^ was chosen. Model basal friction was kept constant.

Simulated runup and inundations at Stromboli were compared with the effects of the 2002 tsunami. Considering that the existing literature presents some differences in describing the 2002’s inundation areas, this data was extracted by comparing the data from Tinti *et al*.^8^, Dall’Osso *et al*.^40^ and the unpublished INGV technical report drawn up soon after the tsunami, as well as considering the local topography. The measured runup on Stromboli was taken from Tinti *et al*.^7^. The detection of tsunami waves, along the coast of the STS, was described by Maramai *et al*.^9^. All collected data were georeferenced, uploaded and analyzed in a GIS environment.

## Results and Discussion

### Landslide kinematics and tsunami generation

**Figure 2:**
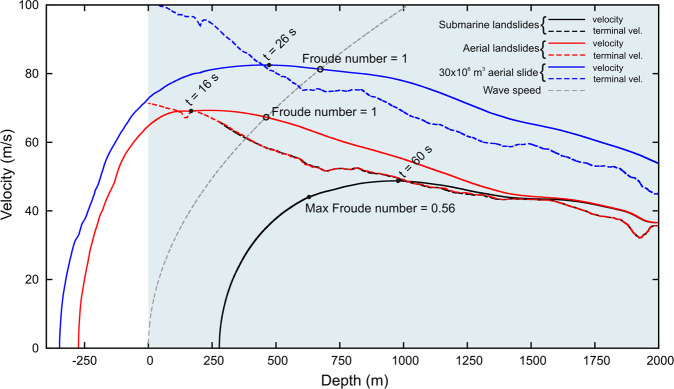
Velocity of the slides vs. depth. Terminal velocity and wave speed are also reported. Slides with same planimetric footprint have the same velocity.

**Figure 3:**
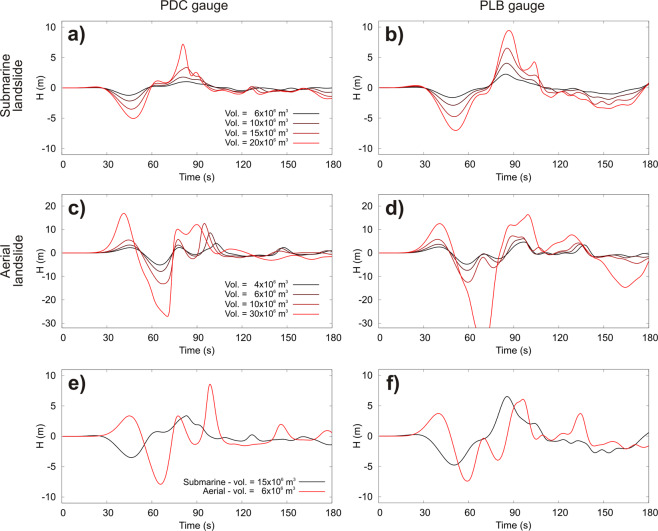
Wave shapes calculated at the monitoring gauges of PDC and PLB. Legends at PDC are also applies for PLB. Comparison between the waves generated by the aerial slide of 7.1 × 10^6^ m^3^ and the subaqueous slides of 17.6 × 10^6^ m^3^ are shown in frames (**e**,**f**).

**Figure 4:**
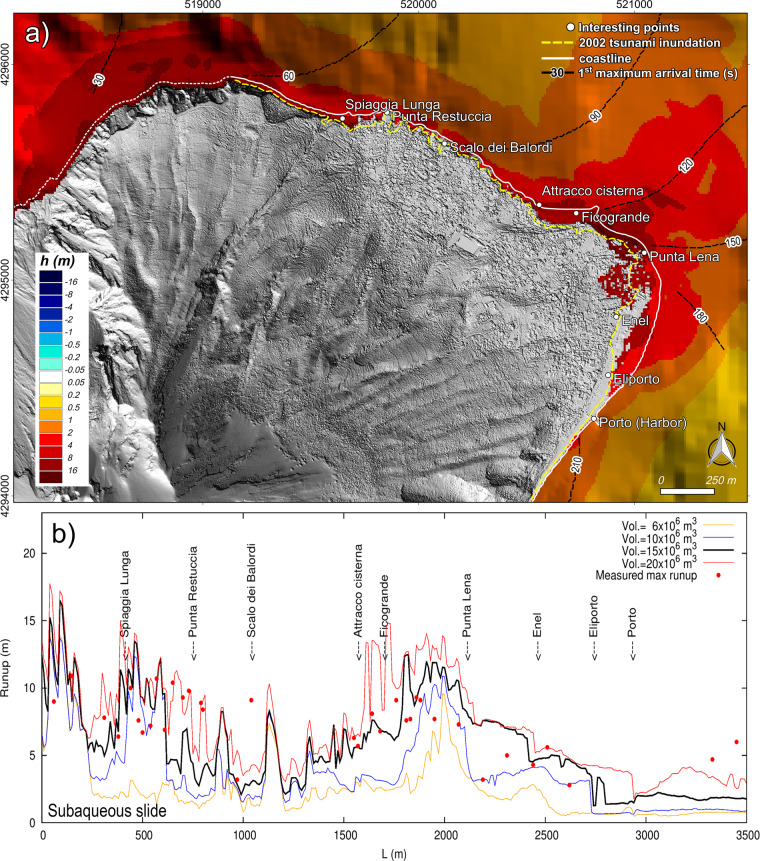
Observed and simulated tsunami effects comparison at Stromboli. (**a**) Stromboli map representing the maximum simulated inundation and wave height for a tsunami caused by a submarine slide along the SdF of 17.6 × 10^6^ m^3^. Map was generated using Quantum GIS 2.8.1 software (https://www.qgis.org). (**b**) Comparison between the 2002 runups observed by Tinti *et al*.^7^ and runups simulated in case of submarine scenarios.

Slide velocities have a trend coherent with that calculated by Tinti *et al*.^7^, i.e. an initial sharp acceleration followed by a phase of slow deceleration (Fig. 2). Slides with the same planimetric footprint have the same velocity despite different volumes. Indeed, the equation of motion described in Eq. (3) depends on the slide geometry only through the term $\frac{{A}_{b}}{{V}_{b}}=\frac{1}{b}$. It follows that slides with the same *b* have the same kinematics. Figure 2 shows that aerial slides with circular footprint have a maximum velocity (*v*_max_) of about 70 m/s, which is reached in 16 s at a depth of 150 m. The 35.3 × 10^6^ m^3^ slide has *v*_max_ = 80 m/s, achieved in 26 s at 500 m bsl. Tinti *et al*.^7^ calculated *v*_max_ for the aerial slides of just under 60 m/s after about 20 s from the trigger. Our submarine slides have *v*_max_ = 49 m/s after 60 s at about 1000 m bsl. Tinti *et al*.^7^ calculated a maximum velocity for the submarine slide of about 40–45 m/s after 40–50 s from the trigger. Figure 2 clearly shows that all velocities tend towards a terminal velocity, which depends on the slope^41,42^. The landslide efficiency in wave generation is controlled by the Froude number (*F*), given by the speed of the landslide relative to the linear shallow water depth wave celerity $\sqrt{gh}$ (wave speed in Fig. 2). An efficient wave generation is given by *F* = 1, which means that the landslide and the tsunami move with the same speed. The further *F* departs from 1, the less efficient the wave generation is. Aerial slides reach *F* = 1 at the intersection between the slide velocity curve and the waves speed parabola. Viceversa, the submarine slides always have *F* less than 1. At equal volume and shape, aerial slides have higher tsunamigenic potentials than submarine ones. *F* = 1 is reached by aerial circular slides after 25 s with a slide velocity of 67 m/s, at 461 m bsl. The Froude number of the 35.3 × 10^6^ m^3^ slide is 1 after 34 s from the trigger at 673 m bsl when the velocity is 80 m/s. The maximum *F* for submarine slides is 0.56, reached after 34 s with a speed of 44 m/s at 616 m bsl. Submarine slides of different volumes, given by different thicknesses in our case, have the same *F*, but the waves generated (Fig. 3) and the effects on the coast are different (Fig. 4). The tsunamigenic potential of a slide does not depend only on *F* but also on slide thickness.

Two monitoring gauges managed by the Laboratory of Experimental Geophysics of the Department of Earth Sciences of the University of Florence (http://lgs.geo.unifi.it/), designed to detect tsunami waves, are located offshore Punta Dei Corvi and Punta Labronzo (Fig. 1). On account of the absence of real data on tsunami waves, at the first instance the alert system necessarily had to be based on simulated waves. Figure 3 shows the wave shapes calculated at the two monitoring gauges for all scenarios. As expected, the first wave of the submarine slides is negative while the aerial slide produces a first positive wave followed by a negative wave with greater amplitude. The first maximum/minimum waves are detected at the gauges after 40–55 s from slide triggering. Figure 3 also highlights a rough proportionality between the amplitude of the first maximum/minimum and the slide volume.

**Figure 5:**
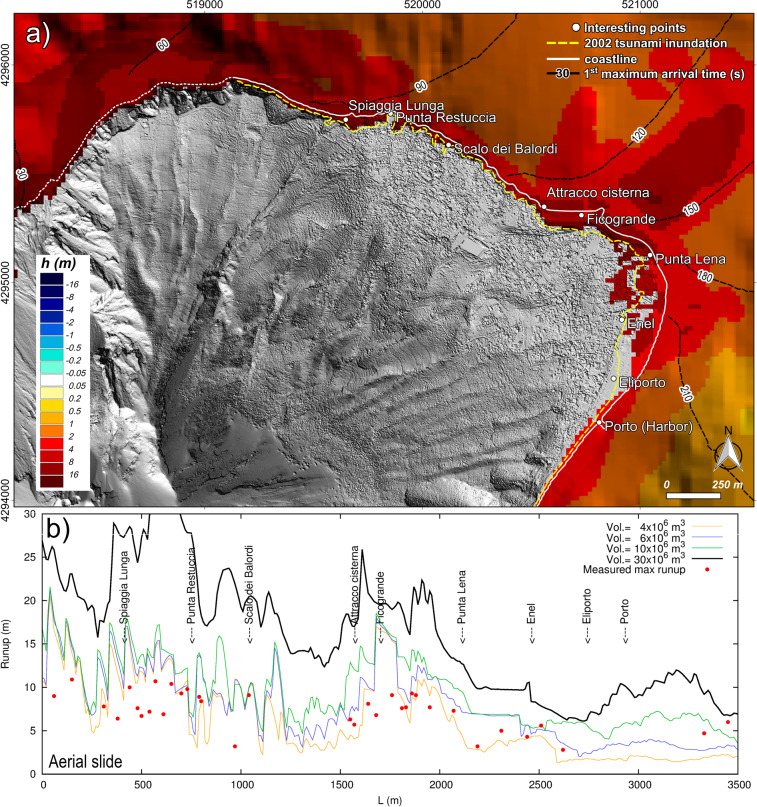
Observed and simulated tsunami effects comparison at Stromboli. (**a**) Stromboli map representing the maximum simulated inundation and wave height for a tsunami caused by an aerial slide along the SdF of 7.1 × 10^6^ m^3^. Map was generated using Quantum GIS 2.8.1 software (https://www.qgis.org). (**b**) Comparison between the 2002 runup observed by Tinti *et al*.^7^ and runups simulated in case of subaerial scenarios.

As reported after the 2002 tsunami, NHWAVE simulations show that although the higher tsunami waves propagate in the northwestward direction, relevant waves are also driven by island bathymetry around Stromboli and they hit inhabited areas (Figs. 4 and 5).

### Proximal impact: Tsunami effect on stromboli island

Figures 4a and 5a illustrate the simulated maximum wave heights and inundation generated by a submarine failure of 17.6 × 10^6^ m^3^ and by an aerial failure of 7.1 × 10^6^ m^3^ on the most inhabited and frequented coast of Stromboli. We represented in Figs. 4a and 5a only these scenarios because they better fit the impact of 2002 tsunami at Stromboli. Figures 4b and 5b show simulated runups versus the 2002 measured runups^8^. Simulations demonstrate that the waves’ impact on the coast is not only directly related to the distance from the source but it is also strongly influenced by the topography and the bathymetry. Starting from the SdF and moving clockwise (towards Stromboli’s Harbor), the runup is initially high on Spiaggia Lunga beach, then it decreases up to Scalo dei Balordi and it drastically increases to a local maximum between Attracco Cisterna and Punta Lena (Figs. 4b and 5b).

In the following paragraph we discuss the simulated tsunami impact regarding only the Northeastern frequented areas between Spiaggia Lunga beach and the Harbor (Porto). Waves generated by the 7.1 × 10^6^ m^3^ subaqueous slide would cause concerns only along the beaches. The maximum penetration (~30 m) and runup (~8 m) are on the beach between Ficogrande and Punta Lena (Fig. 4b). The 11.8 × 10^6^ m^3^ slide causes significant inundation on the main beaches. Just before Punta Lena, waves affect not only the shore but also the residential area. The simulation shows here a maximum penetration of ~110 m and a runup locally over 10 m. It is worth to note that in this area the simulation overestimates the impact of the 2002 event (Fig. 4b). The 17.6 × 10^6^ m^3^ slide scenario fits well the impact of the 2002 tsunami along the north-east coast of Stromboli, both as inland penetration (Fig. 4a) and runup (Fig. 4b). The simulated runup is often over 10 m between Spiaggia Lunga and Punta Restuccia and between Ficogrande and Punta Lena. The maximum penetration is ~250 m at Punta Lena. Compared to the 2002 event, the simulation overestimates the tsunami impact on the inland of Punta Lena and underestimates it on the beach in front of the power plant (Enel, Fig. 4a). Differently, the 23.5 × 10^6^ m^3^ slide impact fits well with the 2002 tsunami effects between Enel and Porto, while it overestimates even more the water inland penetration around Punta Lena (>250 m). The 23.5 × 10^6^ m^3^ scenario shows a stretch of runup just below 15 m between Attracco Cisterna and Punta Lena. The arrival time of the highest waves on the coast is highly fragmented because they are formed as a consequence of waves refraction-diffraction along the rugged coastline. For this reason, in Fig. 4a we report the arrival time of the first positive surface perturbation, which is not affected by interaction with previous waves. In general, the waves’ travel time is quite similar through different scenarios. The first positive wave hits Spiaggia Lunga after just above 1 minute from the slide failure, in 2–3 minutes it reaches Punta Lena and in just under 3.5 minutes arrives at Porto, i.e. about 3 km far from the source. The maximum wave hits Spiaggia Lunga after 2–2.5 minutes, Punta Lena in 5–9 minutes and Porto in about 5 minutes.

At equal volume, aerial slides generate more devastating waves compared to subaqueous ones. The 4.7 × 10^6^ m^3^ slide would already be capable of largely inundating the north-east coast with a runup of 10–11 m (locally even more) between Ficogrande and Punta Lena with a maximum penetration of ~90 m. This runup fits well with the observed 2002 runup but the inundation area is underestimated after Punta Lena. The 7.1 × 10^6^ m^3^ aerial slide has a stronger impact than the subaqueous slide of the same volume. The water inland penetration fits well with 2002 tsunami impact with the exception of the area between Punta Lena and Enel where simulation overestimates it with a maximum penetration reaching ~240 m (Fig. 5a). The runups in the heavily affected area, i.e. between Attracco Cisterna and Punta Lena, range from ~8 to ~18 m (Fig. 5b). The 11.8 × 10^6^ m^3^ aerial slide causes large inundation with maximum runups ranging from 12 to 19 m, again between Attracco Cisterna and Punta Lena. Finally, the 35.3 × 10^6^ m^3^ aerial slide would cause dramatic inundations. Runups higher than 30 m would interest the coast between Spiaggia Lunga and Punta Restuccia. The areas between Attracco Cisterna and Punta Lena would be affected by a runup of over 20 m. As for the arrival of the first waves, similarly to the subaqueous scenarios, the travel time is very similar for the different slide volumes. The first perturbation hits Spiaggia Lunga after ~1.5 minutes from the failure, it reaches Punta Lena about 1–1.5 minute later and arrives at the harbor in under 4 minutes. From the complex pattern of arrival time of the maximum perturbation, we can infer that the highest wave hits Spiaggia Lunga in 2–2.5 minutes, hits Punta Lena in about 3–6 minutes and the harbor in 4–7 minutes.

### Distal impact: Tsunami effect on aeolian arc and south tyrrhenian coasts

**Figure 6:**
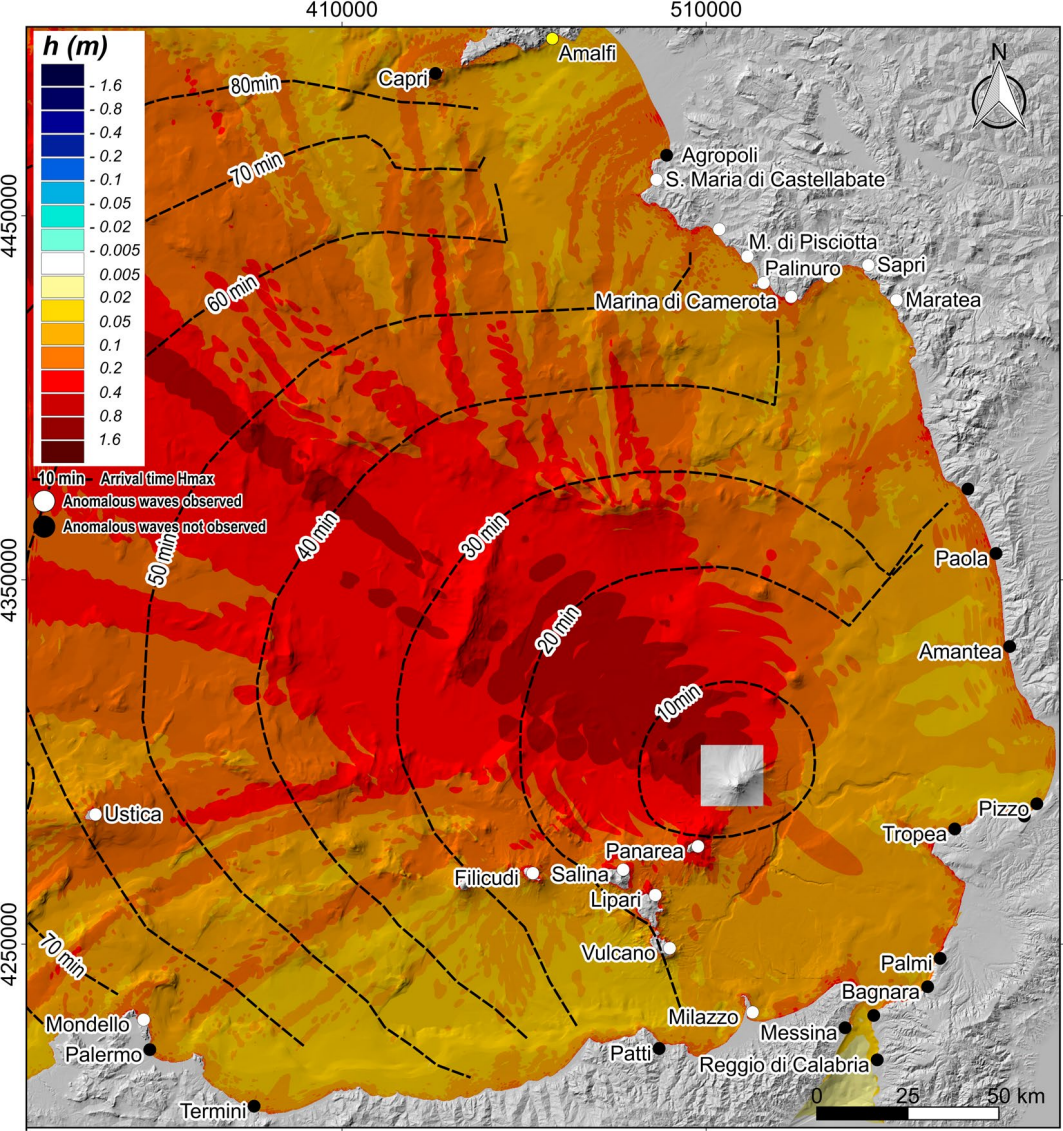
Map illustrating the maximum simulated wave height in the STS caused by a submarine slide of 17.6 × 10^6^ m^3^. Dots represent sites where anomalous waves were observed (white dots) or not observed (black dots) after the 2002 event at Stromboli by Maramai *et al*.^9^. Location of Amalfi is also reported. Map was generated using Quantum GIS 2.8.1 software (https://www.qgis.org).

Tsunami simulations on the extended computational grid were run with the FUNWAVE-TVD model by using as initial condition the wave train generated by 11.8 and 17.6 million submarine slides and 7.1 and 35.3 million aerial slides. From Stromboli, waves trains propagate in any direction impacting the neighboring islands of Aeolian Arc at first and then the remaining Southern Tyrrhenian Coast of Italy. Due to the directional behavior of landslide generated tsunami waves, even coasts very far from the source can be seriously damaged (Fig. 6). Arrival time of the maximum height (and in brackets of the first perturbation) is again similar for all the simulated scenarios. Panarea is impacted in 8–10 (5) minutes; Lipari, Salina and Vulcano are hit in 10–15 (7–8) minutes and in ~30 (13) minutes the whole Aeolian Arc is interested by tsunami waves. In 40–50 (25) minutes waves are offshore of Palinuro and in ~70 (28) minutes offshore of Mondello and Palermo. In ~80 (35) minutes waves arrive at Capri (Fig. 6). Tropea, which is in the opposite direction of the main train propagation, is hit in just under 20 (12) minutes. For the 11.8 × 10^6^ m^3^ subaqueous slide, the simulation shows a maximum wave of about 1 m, 100 m (i.e. grid cell size) offshore of Panarea and a maximum wave of 0.5–1.1 m and 0.8 m off the north coasts of Salina and Lipari, respectively. Off the east coast of Volcano Island, in front of the flat highly inhabited area, the maximum simulated waves are 0.3 m high on average. In the area around Palermo, Mondello is the most impacted area with a maximum wave of 0.3 m. In the rest of the STS, along the coasts of Marina di Camarota, Capri and Tropea, maximum waves reach 0.6 m, 0.2 m and 0.5 m. The 17.6 × 10^6^ m^3^ subaqueous slide would generate maximum waves of 1–1.4 m, 0.6–1.8 m, 1.4 m and 0.5 m for Panarea, Salina, Lipari and Vulcano, respectively. Maximum waves of 0.4 m, 0.8 m, 0.4 m and 0.8 m are calculated along the coasts of Mondello, Marina di Camarota, Capri and Tropea. The 7.1 × 10^6^ m^3^ aerial slide generates wave that reaches 0.8–1.8 m, 0.5–1.2 m, 0.8 m and 0.4 m along the coasts of Panarea, Lipari, Salina and Vulcano. Waves of 0.3 m, 0.6 m, 0.3 m and 0.6 m are calculated offshore of Mondello, Marina di Camarota, Capri and Tropea, respectively. The tsunami generated by a 35.3 × 10^6^ m^3^ aerial slide would severely impacts Panarea with maximum waves up to 4.5 m. Similarly, waves in front of the north coast of Salina and Lipari range from 2 m to 5 m. Waves of 1.5 m can be found in the most vulnerable area of Vulcano. The rest of the Southern Tyrrhenian coasts can be hardly damaged, e.g. waves of 1 m are expected off of Mondello, up to 2.4 m off of Marina di Camarota and 2.2 m off of Tropea. Finally, simulated waves arrive offshore of Capri with a maximum height of 2 m.

### Comparison with previous works

Landslide-generated tsunami at Stromboli was already simulated by using a wave-propagation model based on shallow water equations, initialized by the vertical displacement of the bottom surface caused by the motion of the underwater slide, by Tinti *et al*.^7,43,44^. Tinti *et al*.^43^ simulated a tsunami triggered by a massive landslide of about 1 km^3^ and its effects on Stromboli and along the south-east Tyrrhenian Sea, approximately between Lamezia (Calabria) and Capo d’Orlando (Sicily). This scenario is consistent with that descripted by Rosi *et al*.^4^, although Tinti *et al*.^43^ did not extend their results as far as the Campanian coast. In order to reconstruct the 2002 tsunami at Stromboli, Tinti *et al*.^7^ simulated four scenarios: (i) two submarine slides of 16 × 10^6^ m^3^ volume with different shapes; (ii) one aerial slide with a 5 × 10^6^ m3 volume; and (iii) one slide partly submerged and partly emerged. Tinti *et al*.^44^ simulated tsunamis triggered by slides located outside the SdF scar. The models used by Tinti *et al*.^7,43,44^ calculate neither the runups nor the flooding, thus neglecting the role of local topography. Where inland topography plays a major role in assessing the tsunami impact, this code may not be appropriate^7^, possibly leading to an underestimation/overestimation of the tsunami hazard and risk assessment. Although the NHWAVE code used here treats the slide as a rigid body, which is a rougher approximation than that of Tinti *et al*.^7^, it calculates the runup and the inland flooding taking into account the role of topography in the tsunami impact. 16 × 10^6^ m^3^ subaqueous scenarios and 5 × 10^6^ m^3^ subaerial scenarios of Tinti *et al*.^7^ can be compared with our scenarios of similar volume, i.e. the 17.6 × 10^6^ m^3^ subaqueous slide and the 7.1 × 10^6^ m^3^ subaerial slide, respectively. The travel times of the first perturbations are in agreement: for both simulations Punta Lena is affected by the tsunami after 2.5–3 minutes (Fig. 4) and Panarea is hit after 5–6 minutes. Tinti *et al*.^7^ show the offshore shapes of tsunami waves at several computational tide gauges, two of which are not far from PDC and PLB locations, although closer to the coast (Fig. 1). Maximum tsunami waves of the subaqueous scenario of Tinti *et al*.^7^ are higher, compared to our simulation. The wave peaks of Tinti *et al*.^7^ are above 10 m at Punta Labronzo and above 5 meters at Punta Chiappe, not far from PDC (Fig. 1). In this work, the maximum waves at PDC and PLB are around 4 and 6 m, respectively (Fig. 2). This difference can be easily ascribed to sampling point locations closer to the coast in the case of Tinti *et al*.^7^. The “extreme water elevation” of Tinti *et al*.^7^ and the here simulated runups of Fig. 4 show a common trend, in spite of a general underestimation of the tsunami effects by Tinti *et al*.^7^. These differences can be partially ascribed to the role of topography on the tsunami impact on the coast. For example, in our simulation Punta Lena results as one of the most exposed areas (Fig. 4). This area can be affected by tsunami waves coming from north, east and south-east. This cumulative effect is taken into account in our simulation. Field data confirm that Punta Lena is particularly exposed to tsunami flooding (Fig. 4a, yellow line). As concerns the aerial slide, the maximum waves sampled by Tinti *et al*.^7^ in the computed tide-gauges nearest to PDC and PLB are about 6 m, while in our simulation they are 8 m at PDC and just below 6 m at PLB. The maximum “synthetic runup” in Tinti *et al*.^7^ calculated for the 5 × 10^6^ m^3^ slide generally underestimates the measured runup. The maximum “synthetic runup” far from the SdF is in Ficograde with a value of about 7 m. Our 7.1 × 10^6^ m^3^ slide simulation generally overestimates the runups, which are better fitted by the 4.7 × 10^6^ m^3^ scenario (Fig. 5b).

Tinti *et al*.^43^ simulated a tsunami triggered by a massive landslide of about 1 km^3^. Slide peak velocity was about 65 m/s. After 120 s, the first perturbation went past Punta Lena where a “synthetic runup” of about 35 m was calculated. A first perturbation reached Lipari in 400 s and then Sicily in 800 s. The highest extreme water elevation in Calabria was about 7.5 m east to Tropea, which was hit 13 minutes after the slide failure. Our worst scenario was triggered by an aerial slide of 35.3 × 10^6^ m^3^, and it reaches a peak velocity of 80 m/s (Fig. 2) and its first perturbation hit Punta Lena in 2.5 minutes with a runup of 20 m. The maximum perturbation hits the coast to the east of Tropea in just above 15 minutes with 2 m high waves, while the first perturbation arrived after 13 minutes, consistent with Tinti *et al*.^43^. The location of the trenches excavated by Rosi *et al*.^4^ would have been inundated even by a tsunami triggered by a 35.3 × 10^6^ m^3^ slide. This slide would also cause waves of ca. 0.5 m offshore Amalfi. Despite difference in the slide volume, this work provides a first analysis of the area that might have been impacted by a massive event like those proposed in Rosi *et al*.^4^.

## Conclusion

The NHWAVE model, with a modified implementation of the slide motion, was used to simulate: i) tsunami generation by aerial and submarine slides along the SdF with various volumes; ii) the waves propagation and impact on the northeastern coasts of Stromboli. NHWAVE solution was then input to the FUNWAVE-TVD model in order to simulate tsunami waves propagation in the STS. NHWAVE is capable of simulating landslide tsunami generation, propagation and impact. In the case of tsunami simulations at Stromboli, NHWAVE had to be adapted to account for aerial landslides and for subaqueous curvilinear trajectories. It also requires huge calculation resources and time, limiting its use both for (almost) real-time purposes and for simulating wave propagation over large computational domains as the STS is. Moreover initialization of the depth-averaged code FUNWAVE-TVD with the output of the multilayer NHWAVE is affected by noise and instabilities. Finally, in order to calculate significant runups, it is mandatory to have a topo-bathymetric grid of proper accuracy and resolution.

The following main conclusions can be drawn from this work:

- At equal volume, aerial slides generate higher waves and runup than the submarine ones (Figs. 3, 4b and 5b). Indeed, at equal volume (i.e. equal thickness in our simulation) the aerial slides go through the maximum tsunamigenic Froude value of 1, while the submarine slides, in our case, always stay under *F* = 0.56 (Fig. 2). Although the undersea part of the Sciara del Fuoco is more unstable than the aerial one, the small tsunamis triggered by the PDCs occurred on 3 July and 28 August 2019 shifted the attention towards subaerial tsunami source. As expected, the aerial slide produces a first positive wave; on the contrary the first wave of the submarine slide is negative (Fig. 3);
- By comparing observed and simulated data, we estimate that the 2002 Stromboli tsunami damages could have been generated either by a subaqueous failure of about a 17.6–23.5 × 10^6^ m^3^ along the SdF or a subaerial failure of about 4.7–7.1 × 10^6^ m^3^ (Figs. 4 and 5). These results are in general agreement with those of Tinti *et al*.^7^;
- There is no linear correlation between the tsunami effects and the distance from the source. Bathymetry is very important and can dramatically increase the waves heights locally^7,14^. Topography is also important because it determines the inundation area. Where inland topography plays a major role in the tsunami impact, it is necessary to calculate runups and inland penetration to avoid underestimation/overestimation in assessing tsunami hazard and risk;
- The most hazardous inhabited area at Stromboli is the stretch of coast comprised between Attracco Cisterna and Punta Lena. This can be related both to the convex saddles between Stromboli and Strombolicchio and the coastal topography (Figs. 4 and 5);
- The first tsunami waves generated along the SdF are able to reach the first populated beaches in just over 1 minute and to hit the Stromboli harbor in just under 3.5 minutes. Maximum waves arrive to Panarea in just over 8–10 minutes and in 30 minutes go past the Aeolian Arc. In 1 hour and 20 minutes, maximum waves travel through the whole STS arriving offshore of Capri;
- Several factors, not all related to the source, influence how strong the impact of the tsunami waves can be. In our case, the directional behavior of a landslide-generated tsunami, the position of the coast in regard to the source, as well as the bathymetry, explain the high waves arriving offshore of Marina di Camerota (Fig. 6), about 130 km from the source, and can also explain the zoned distribution of sites (Fig. 6) where the 2002 tsunami waves were observed^9^.

This study can improve the understanding of the triggering mechanism and the wave impact of future tsunami at Stromboli as in the case of the small tsunami occurred during the 3 July 2019 and 28 August 2019 paroxysms.
