# Author Correction: Numerical simulation of the tsunamis generated by the Sciara del Fuoco landslides (Stromboli Island, Italy)

**DOI:** 10.1038/s41598-021-99826-4

**Published:** 2021-11-02

**Authors:** A. Fornaciai, M. Favalli, L. Nannipieri

**Affiliations:** Istituto Nazionale di Geofisica e Vulcanologia, Sezione di Pisa, Pisa, Italy

Correction to: *Scientific Reports* 10.1038/s41598-019-54949-7, published online 06 December 2019

The original version of this Article contained errors in volume calculations as a result of an error in the formula (2) of Enet and Grill (2007) and later corrected in Schambach et al. (2019).

In the Abstract,

“We simulated the following scenarios: (i) the tsunami runup, inland inundation and wave propagation at Stromboli triggered by submarine landslides with volumes of 6, 10, 15 and 20×10^6^ m^3^ and subaerial landslides with volumes of 4, 6, 10 and 30×10^6^ m^3^; (ii) tsunami propagation in the STS triggered by submarine landslides with volumes of 10 and 15×10^6^ m^3^ and by subaerial landslides with volumes of 6 and 30×10^6^ m^3^. We estimate that the damages of the last relevant tsunami at Stromboli, which occurred in 2002, could have been generated either by a subaqueous failure of about 15–20×10^6^ m^3^ along the SdF or/and a subaerial failure of about 4–6×10^6^ m^3^.”

now reads:

“We simulated the following scenarios: (i) the tsunami runup, inland inundation and wave propagation at Stromboli triggered by submarine landslides with volumes of 7.1, 11.8, 17.6 and 23.5× 10^6^ m^3^ and subaerial landslides with volumes of 4.7, 7.1, 11.8 and 35.3× 10^6^ m^3^; (ii) tsunami propagation in the STS triggered by submarine landslides with volumes of 11.8 and 17.6× 10^6^ m^3^ and by subaerial landslides with volumes of 7.1 and 35.3× 10^6^ m^3^. We estimate that the damages of the last relevant tsunami at Stromboli, which occurred in 2002, could have been generated either by a subaqueous failure of about 17.6-23.5 × 10^6^ m^3^ along the SdF or/and a subaerial failure of about 4.7-7.1× 10^6^ m^3^.”

In the Tsunami Simulation section, under the subheading ‘Slide scenarios and 2002 field data’,

“Four slide scenarios were considered for both subaerial and submarine mass failure. Based on volumes inferred by Chiocci *et al*.^33^, the extreme case scenarios hypothesized by Tommasi *et al*.^34^ for the 2002 tsunami and with the aim of identifying tsunami wave impact scenarios in the STS coasts, we simulated tsunamis generated by submarine slides of 6, 10, 15 and 20 × 10^6^ m^3^ and subaerial slides of 4, 6, 10 and 30 × 10^6^ m^3^.”

now reads:

“Four slide scenarios were considered for both subaerial and submarine mass failure. Based on volumes inferred by Chiocci *et al*.^33^, the extreme case scenarios hypothesized by Tommasi *et al*.^34^ for the 2002 tsunami and with the aim of identifying tsunami wave impact scenarios in the STS coasts, we simulated tsunamis generated by submarine slides of 7.1, 11.8, 17.6 and 23.5 × 10^6^ m^3^ and subaerial slides of 4.7, 7.1, 11.8 and 35.3 × 10^6^ m^3^.”

Under the same subheading,

“Aside from the worst case (i.e. 30 × 10^6^ m^3^), the slides in all scenarios had a circular footprint with a radius of 670 m (Fig. 1). The 30 × 10^6^ m^3^ slide had an elliptical footprint with the major axis (1300 m) following the inclination of SdF and the minor one (600 m) perpendicular to it (Fig. 1). Submarine slides centers coordinates were: x = 517563, y = 4295449 and z =  − 293 m. Centers coordinates of sub-aerial slides with circular footprints were: x = 518054, y = 4294622 and z = 250 m. Due to its size and footprint, the center coordinates for the 30 × 10^6^ m^3^ slide were: x = 518186, y = 4294600 and z = 350 m (Fig. 1). Coordinates are in WGS84, UTM 33 reference system. To account for the different volumes, the slide thickness changed for every scenario, i.e. for the 4, 6, 10, 15, 20 and 30 × 10^6^ m^3^ cases, the maximum thicknesses were respectively 29.9, 45.0, 74.7, 112.0, 194.4 and m.149.4 Considering the slide as rigid body of volcanoclastic material, a constant density of 2600 kg/m^3^ was chosen.

now reads:

“Aside from the worst case (i.e. 35.3 × 10^6^ m^3^), the slides in all scenarios had a circular footprint with a radius of 670 m (Fig. 1). The 35.3 × 10^6^ m^3^ slide had an elliptical footprint with the major axis (1300 m) following the inclination of SdF and the minor one (600 m) perpendicular to it (Fig. 1). Submarine slides centers coordinates were: x = 517563, y = 4295449 and z = −293 m. Centers coordinates of sub-aerial slides with circular footprints were: x = 518054, y = 4294622 and z = 250 m. Due to its size and footprint, the center coordinates for the 35.3 × 10^6^ m^3^ slide were: x = 518186, y= 4294600 and z = 350 m (Fig. 1). Coordinates are in WGS84, UTM 33 reference system. To account for the different volumes, the slide thickness changed for every scenario, i.e. for the 4.7, 7.1, 11.8, 17.6, 23.5 and 35.3 × 10^6^ m^3^ cases, the maximum thicknesses were respectively 29.9, 45.0, 74.7, 112.0, 194.4 and 149.4 m. Considering the slide as rigid body of volcanoclastic material, a constant density of 2600 kg/m^3^ was chosen.”

In the Results and Discussion, under the subheading ‘Landslide kinematics and tsunami generation’,

“The 30 × 10^6^ m^3^ slide has v_max_ = 80 m/s, achieved in 26 s at 500 m bsl. Tinti *et al*.^7^ calculated v_max_ for the aerial slides of just under 60 m/s after about 20 s from the trigger.”

now reads*:*

“The 35.3 × 10^6^ m^3^ slide has v_max_ = 80 m/s, achieved in 26 s at 500 m bsl. Tinti *et al*.^7^ calculated v_max_ for the aerial slides of just under 60 m/s after about 20 s from the trigger.”

Under the same subheading,

“The Froude number of the 30 × 10^6^ m^3^ slide is 1 after 34 s from the trigger at 673 m bsl when the velocity is 80 m/s.”

now reads:

“The Froude number of the 35.3 × 10^6^ m^3^ slide is 1 after 34 s from the trigger at 673 m bsl when the velocity is 80 m/s.”

Under the subheading ‘Proximal impact: Tsunami effect on stromboli island’,

“Figures 4a and 5a illustrate the simulated maximum wave heights and inundation generated by a submarine failure of 15 × 10^6^ m^3^ and by an aerial failure of 6 × 10^6^ m^3^ on the most inhabited and frequented coast of Stromboli.”

now reads:

“Figures 4a and 5a illustrate the simulated maximum wave heights and inundation generated by a submarine failure of 17.6 × 10^6^ m^3^ and by an aerial failure of 7.1 × 10^6^ m^3^ on the most inhabited and frequented coast of Stromboli.”

Under the same subheading,

“Waves generated by the 6 × 10^6^ m^3^ subaqueous slide would cause concerns only along the beaches. The maximum penetration (~30 m) and runup (~8 m) are on the beach between Ficogrande and Punta Lena (Fig. 4b). The 10 × 10^6^ m^3^ slide causes significant inundation on the main beaches. Just before Punta Lena, waves affect not only the shore but also the residential area. The simulation shows here a maximum penetration of ~110 m and a runup locally over 10 m. It is worth to note that in this area the simulation overestimates the impact of the 2002 event (Fig. 4b). The 15 × 10^6^ m^3^ slide scenario fits well the impact of the 2002 tsunami along the north-east coast of Stromboli, both as inland penetration (Fig. 4a) and runup (Fig. 4b). The simulated runup is often over 10 m between Spiaggia Lunga and Punta Restuccia and between Ficogrande and Punta Lena. The maximum penetration is ~250 m at Punta Lena. Compared to the 2002 event, the simulation overestimates the tsunami impact on the inland of Punta Lena and underestimates it on the beach in front of the power plant (Enel, Fig. 4a). Differently, the 20 × 10^6^ m^3^ slide impact fits well with the 2002 tsunami effects between Enel and Porto, while it overestimates even more the water inland penetration around Punta Lena (>250 m). The 20 × 10^6^ m^3^ scenario shows a stretch of runup just below 15 m between Attracco Cisterna and Punta Lena.”

now reads:

“Waves generated by the 7.1 × 10^6^ m^3^ subaqueous slide would cause concerns only along the beaches. The maximum penetration (~30 m) and runup (~8 m) are on the beach between Ficogrande and Punta Lena (Fig. 4b). The 11.8 × 10^6^ m^3^ slide causes significant inundation on the main beaches. Just before Punta Lena, waves affect not only the shore but also the residential area. The simulation shows here a maximum penetration of ~110 m and a runup locally over 10 m. It is worth to note that in this area the simulation overestimates the impact of the 2002 event (Fig. 4b). The 17.6 × 10^6^ m^3^ slide scenario fits well the impact of the 2002 tsunami along the north-east coast of Stromboli, both as inland penetration (Fig. 4a) and runup (Fig. 4b). The simulated runup is often over 10 m between Spiaggia Lunga and Punta Restuccia and between Ficogrande and Punta Lena. The maximum penetration is ~250 m at Punta Lena. Compared to the 2002 event, the simulation overestimates the tsunami impact on the inland of Punta Lena and underestimates it on the beach in front of the power plant (Enel, Fig. 4a). Differently, the 23.5 × 10^6^ m^3^ slide impact fits well with the 2002 tsunami effects between Enel and Porto, while it overestimates even more the water inland penetration around Punta Lena (>250 m). The 23.5 × 10^6^ m^3^ scenario shows a stretch of runup just below 15 m between Attracco Cisterna and Punta Lena.”

And,

“The 4 × 10^6^ m^3^ slide would already be capable of largely inundating the north-east coast with a runup of 10–11 m (locally even more) between Ficogrande and Punta Lena with a maximum penetration of ~90 m. This runup fits well with the observed 2002 runup but the inundation area is underestimated after Punta Lena. The 6 × 10^6^ m^3^ aerial slide has a stronger impact than the subaqueous slide of the same volume. The water inland penetration fits well with 2002 tsunami impact with the exception of the area between Punta Lena and Enel where simulation overestimates it with a maximum penetration reaching ~240 m (Fig. 5a). The runups in the heavily affected area, i.e. between Attracco Cisterna and Punta Lena, range from ~8 to ~18 m (Fig. 5b). The 11.8 × 10^6^ m^3^ aerial slide causes large inundation with maximum runups ranging from 12 to 19 m, again between Attracco Cisterna and Punta Lena. Finally, the 30 × 10^6^ m^3^ aerial slide would cause dramatic inundations.”

now reads:

“The 4.7 × 10^6^ m^3^ slide would already be capable of largely inundating the north-east coast with a runup of 10–11 m (locally even more) between Ficogrande and Punta Lena with a maximum penetration of ~90 m. This runup fits well with the observed 2002 runup but the inundation area is underestimated after Punta Lena. The 7.1 × 10^6^ m^3^ aerial slide has a stronger impact than the subaqueous slide of the same volume. The water inland penetration fits well with 2002 tsunami impact with the exception of the area between Punta Lena and Enel where simulation overestimates it with a maximum penetration reaching ~240 m (Fig. 5a). The runups in the heavily affected area, i.e. between Attracco Cisterna and Punta Lena, range from ~8 to ~18 m (Fig. 5b). The 11.8 × 10^6^ m^3^ aerial slide causes large inundation with maximum runups ranging from 12 to 19 m, again between Attracco Cisterna and Punta Lena. Finally, the 35.3 × 10^6^ m^3^ aerial slide would cause dramatic inundations.”

Under the subheading ‘Distal impact: Tsunami effect on aeolian arc and south tyrrhenian coasts’,

“Tsunami simulations on the extended computational grid were run with the FUNWAVE-TVD model by using as initial condition the wave train generated by 10 and 15 million submarine slides and 6 and 30 million aerial slides.”

now reads:

“Tsunami simulations on the extended computational grid were run with the FUNWAVE-TVD model by using as initial condition the wave train generated by 11.8 and 17.6 million submarine slides and 7.1 and 35.3 million aerial slides.”

Under the same subheading,

“For the 10 × 10^6^ m^3^ subaqueous slide, the simulation shows a maximum wave of about 1 m, 100 m (i.e. grid cell size) offshore of Panarea and a maximum wave of 0.5–1.1 m and 0.8 m off the north coasts of Salina and Lipari, respectively.”

now reads:

“For the 11.8 × 10^6^ m^3^ subaqueous slide, the simulation shows a maximum wave of about 1 m, 100 m (i.e. grid cell size) offshore of Panarea and a maximum wave of 0.5–1.1 m and 0.8 m off the north coasts of Salina and Lipari, respectively.”

And,

“The 15 × 10^6^ m^3^ subaqueous slide would generate maximum waves of 1–1.4 m, 0.6–1.8 m, 1.4 m and 0.5 m for Panarea, Salina, Lipari and Vulcano, respectively.”

now reads:

“The 17.6 × 10^6^ m^3^ subaqueous slide would generate maximum waves of 1–1.4 m, 0.6–1.8 m, 1.4 m and 0.5 m for Panarea, Salina, Lipari and Vulcano, respectively.”

And,

“The 6 × 10^6^ m^3^ aerial slide generates wave that reaches 0.8–1.8 m, 0.5–1.2 m, 0.8 m and 0.4 m along the coasts of Panarea, Lipari, Salina and Vulcano.”

now reads:

“The 7.1 × 10^6^ m^3^ aerial slide generates wave that reaches 0.8–1.8 m, 0.5–1.2 m, 0.8 m and 0.4 m along the coasts of Panarea, Lipari, Salina and Vulcano.”

And,

“The tsunami generated by a 30 ×10^6^ m^3^ aerial slide would severely impact Panarea with maximum waves up to 4.5 m.”

now reads:

“The tsunami generated by a 35.3 ×10^6^ m^3^ aerial slide would severely impact Panarea with maximum waves up to 4.5 m.”

Under the subheading ‘Comparison with previous works’,

“Although the NHWAVE code used here treats the slide as a rigid body, which is a rougher approximation than that of Tinti *et al*.^7^, it calculates the runup and the inland flooding taking into account the role of topography in the tsunami impact. 16 × 10^6^ m^3^ subaqueous scenarios and 5 × 10^6^ m^3^ subaerial scenarios of Tinti *et al.*^7^ can be compared with our scenarios of similar volume, i.e. the 15 × 10^6^ m^3^ subaqueous slide and the 6 ×10^6^ m^3^ subaerial slide, respectively.”

now reads:

“Although the NHWAVE code used here treats the slide as a rigid body, which is a rougher approximation than that of Tinti *et al*.^7^, it calculates the runup and the inland flooding taking into account the role of topography in the tsunami impact. 16 × 10^6^ m^3^ subaqueous scenarios and 5 × 10^6^ m^3^ subaerial scenarios of Tinti *et al.*^7^ can be compared with our scenarios of similar volume, i.e. the 17.6 × 10^6^ m^3^ subaqueous slide and the 7.1 ×10^6^ m^3^ subaerial slide, respectively.”

Under the same subheading,

“Our 6 × 10^6^ m^3^ slide simulation generally overestimates the runups, which are better fitted by the 4 × 10^6^ m^3^ scenario (Fig. 5b).”

now reads:

“Our 7.1 × 10^6^ m^3^ slide simulation generally overestimates the runups, which are better fitted by the 4.7 × 10^6^ m^3^ scenario (Fig. 5b).”

And,

“Our worst scenario was triggered by an aerial slide of 30 × 10^6^ m^3^, and it reaches a peak velocity of 80 m/s (Fig. 2) and its first perturbation hit Punta Lena in 2.5 minutes with a runup of 20 m.

now reads:

“Our worst scenario was triggered by an aerial slide of 35.3 × 10^6^ m^3^, and it reaches a peak velocity of 80 m/s (Fig. 2) and its first perturbation hit Punta Lena in 2.5 minutes with a runup of 20 m.

And,

“The location of the trenches excavated by Rosi *et al*.^4^ would have been inundated even by a tsunami triggered by a 30 × 10^6^ m^3^ slide.”

now reads:

“The location of the trenches excavated by Rosi *et al.*^4^ would have been inundated even by a tsunami triggered by a 35.3 × 10^6^ m^3^ slide.”

In the Conclusion, the second bullet point,

“By comparing observed and simulated data, we estimate that the 2002 Stromboli tsunami damages could have been generated either by a subaqueous failure of about a 15-20 × 10^6^ m^3^ along the SdF or a subaerial failure of about 4-6 × 10^6^ m^3^ (Figs. 4 and 5).”

now reads:

“By comparing observed and simulated data, we estimate that the 2002 Stromboli tsunami damages could have been generated either by a subaqueous failure of about a 17.6-23.5 × 10^6^ m^3^ along the SdF or a subaerial failure of about 4.7-7.1 × 10^6^ m^3^ (Figs. 4 and 5).”

Finally, as a result of the changes, Figures 2, 3, 4 and 5, and the legends of Figures 3, 4, 5 and 6 were incorrect.

In Figure 2, the key label “35.3 × 10^6^ aerial slide” was incorrectly given as “30 × 10^6^ m^3^ aerial slide.”

In Figure 3a,c,e, Figure 4b, and Figure. 5b, the key labels for “Vol” were incorrectly given.

The legend for Figure 3,

“Wave shapes calculated at the monitoring gauges of PDC and PLB. Legends at PDC are also applies for PLB. Comparison between the waves generated by the aerial slide of 6 × 10^6^ m^3^ and the subaqueous slides of 15 × 10^6^ m^3^ are shown in frames (**e**,**f**).”

now reads:

“Wave shapes calculated at the monitoring gauges of PDC and PLB. Legends at PDC are also applies for PLB. Comparison between the waves generated by the aerial slide of 7.1 × 10^6^ m^3^ and the subaqueous slides of 17.6 × 10^6^ m^3^ are shown in frames (**e**,**f**).”

The legend for Figure 4,

“Observed and simulated tsunami effects comparison at Stromboli. (**a**) Stromboli map representing the maximum simulated inundation and wave height for a tsunami caused by a submarine slide along the SdF of 15 × 10^6^ m^3^. Map was generated using Quantum GIS 2.8.1 software (https://www.qgis.org). (**b**) Comparison between the 2002 runups observed by Tinti *et al*.^7^ and runups simulated in case of submarine scenarios.”

now reads:

“Observed and simulated tsunami effects comparison at Stromboli. (**a**) Stromboli map representing the maximum simulated inundation and wave height for a tsunami caused by a submarine slide along the SdF of 17.6 × 10^6^ m^3^. Map was generated using Quantum GIS 2.8.1 software (https://www.qgis.org). (**b**) Comparison between the 2002 runups observed by Tinti *et al.*^7^ and runups simulated in case of submarine scenarios.”

The legend for Figure 5,

“Observed and simulated tsunami effects comparison at Stromboli. (**a**) Stromboli map representing the maximum simulated inundation and wave height for a tsunami caused by an aerial slide along the SdF of 6 × 10^6^ m^3^. Map was generated using Quantum GIS 2.8.1 software (https://www.qgis.org). (**b**) Comparison between the 2002 runup observed by Tinti *et al*.^7^ and runups simulated in case of subaerial scenarios.”

now reads:

“Observed and simulated tsunami effects comparison at Stromboli. (**a**) Stromboli map representing the maximum simulated inundation and wave height for a tsunami caused by an aerial slide along the SdF of 7.1 × 10^6^ m^3^. Map was generated using Quantum GIS 2.8.1 software (https://www.qgis.org). (**b**) Comparison between the 2002 runup observed by Tinti *et al.*^7^ and runups simulated in case of subaerial scenarios.”

The legend for Figure 6,

“Map illustrating the maximum simulated wave height in the STS caused by a submarine slide of 15 × 10^6^m^3^.”

now reads:

“Map illustrating the maximum simulated wave height in the STS caused by a submarine slide of 17.6 × 10^6^m^3^.”

The original Figures 2, 3, 4 and 5 and accompanying legends appear below.

**Figure 2:**
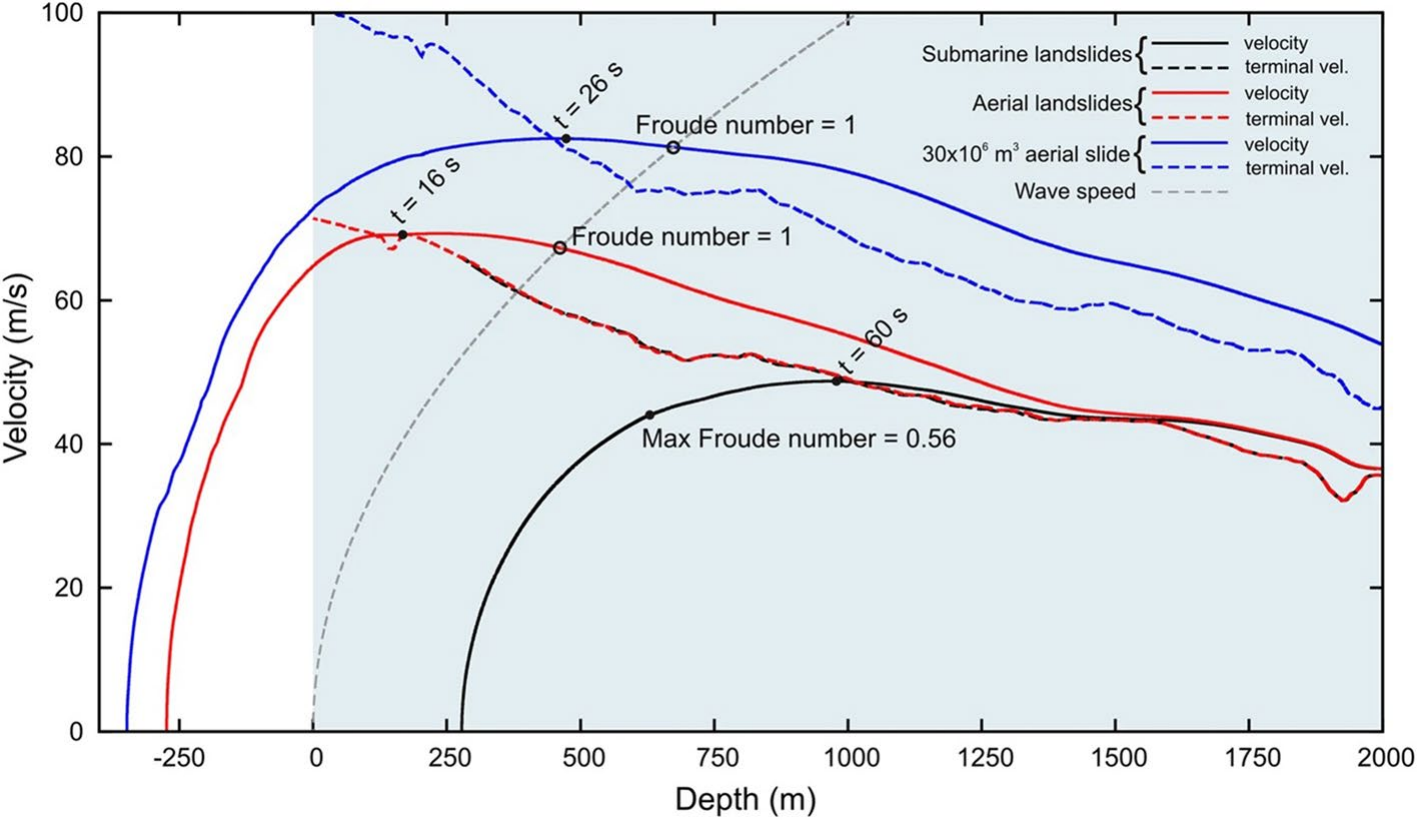
Velocity of the slides vs. depth. Terminal velocity and wave speed are also reported. Slides with same planimetric footprint have the same velocity.

**Figure 3:**
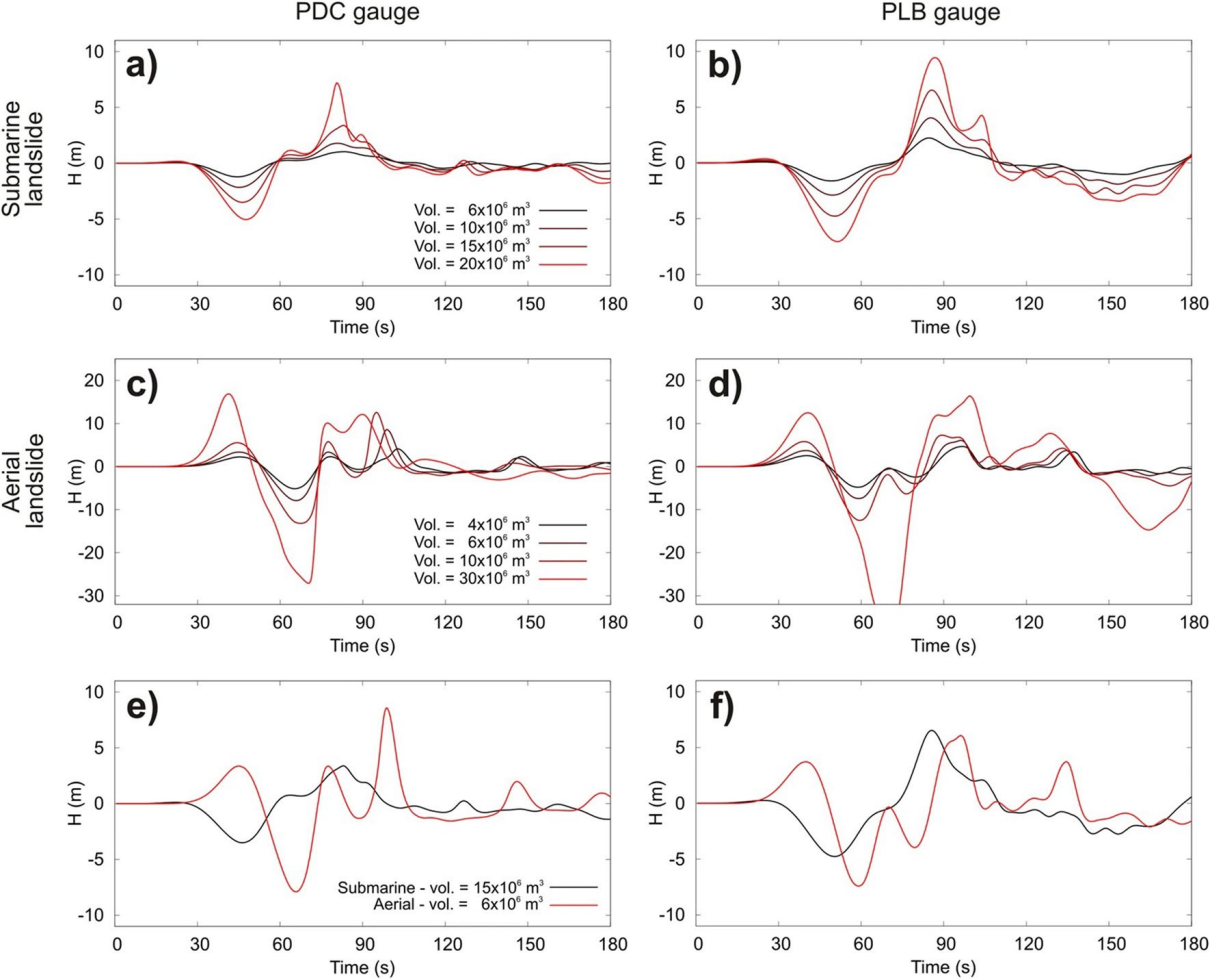
Wave shapes calculated at the monitoring gauges of PDC and PLB. Legends at PDC are also applies for PLB. Comparison between the waves generated by the aerial slide of 6 × 10^6^ m^3^ and the subaqueous slides of 15 × 10^6^ m^3^ are shown in frames (**e**,**f**).

**Figure 4:**
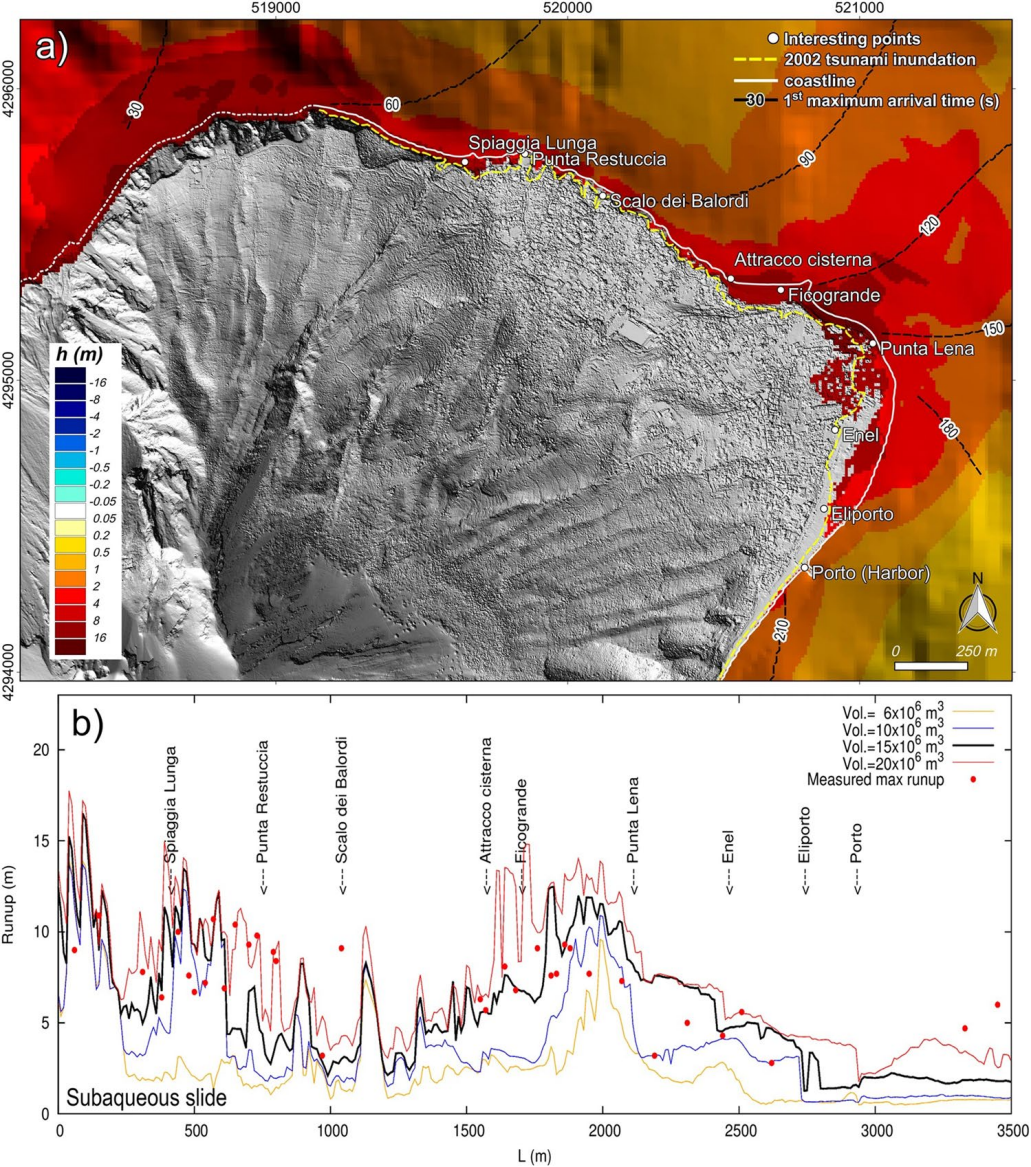
Observed and simulated tsunami effects comparison at Stromboli. (**a**) Stromboli map representing the maximum simulated inundation and wave height for a tsunami caused by a submarine slide along the SdF of 15 × 10^6^ m^3^. Map was generated using Quantum GIS 2.8.1 software (https://www.qgis.org). (**b**) Comparison between the 2002 runups observed by Tinti et al.^7^ and runups simulated in case of submarine scenarios.

**Figure 5:**
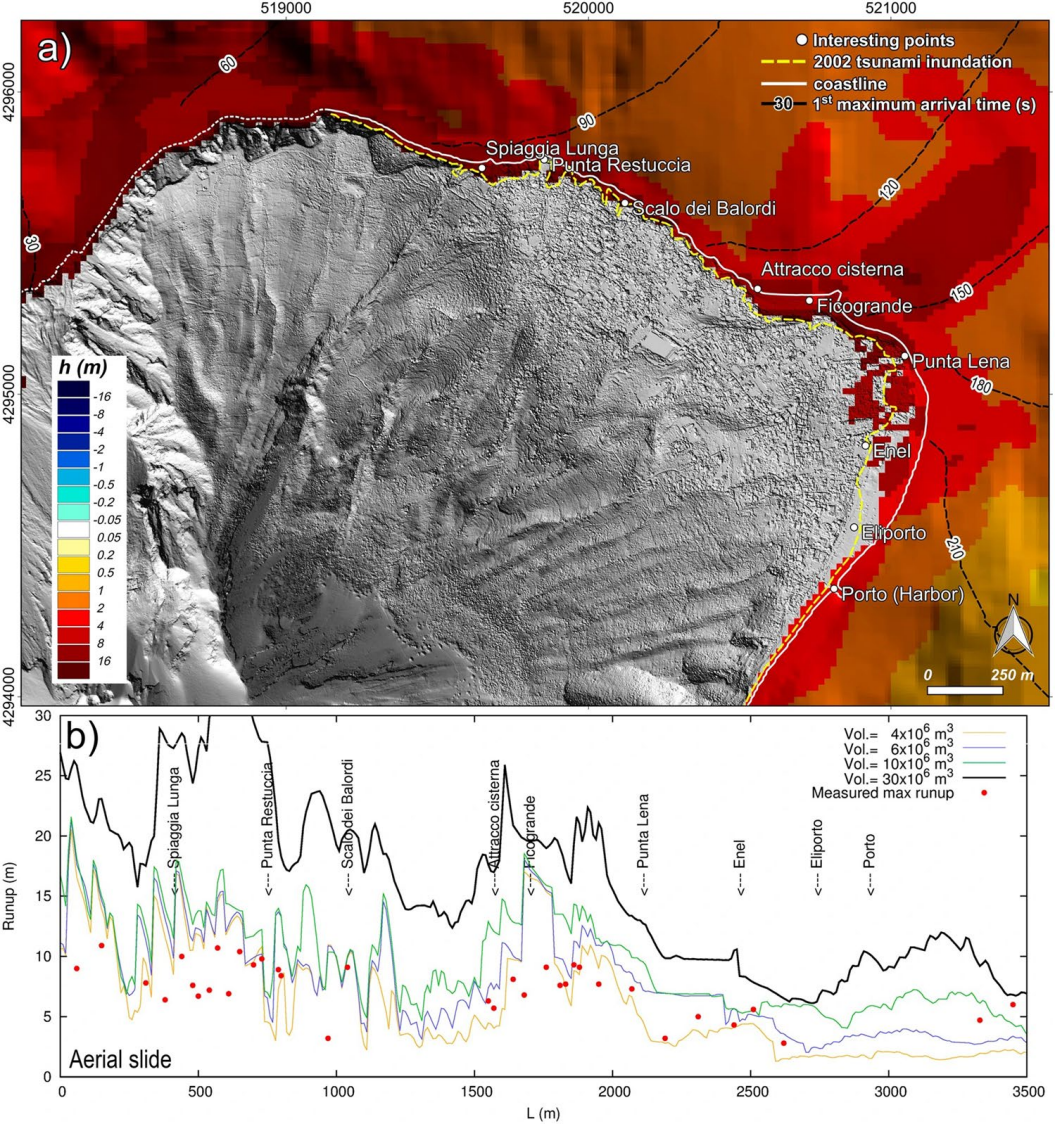
Observed and simulated tsunami effects comparison at Stromboli. (**a**) Stromboli map representing the maximum simulated inundation and wave height for a tsunami caused by an aerial slide along the SdF of 6 × 10^6^ m^3^. Map was generated using Quantum GIS 2.8.1 software (https://www.qgis.org). (**b**) Comparison between the 2002 runup observed by Tinti et al.^7^ and runups simulated in case of subaerial scenarios.

The original Article has been corrected.

